# Loss of ARHGEF6 Causes Hair Cell Stereocilia Deficits and Hearing Loss in Mice

**DOI:** 10.3389/fnmol.2018.00362

**Published:** 2018-10-02

**Authors:** Chengwen Zhu, Cheng Cheng, Yanfei Wang, Waqas Muhammad, Shuang Liu, Weijie Zhu, Buwei Shao, Zhong Zhang, Xiaoqian Yan, Qingqing He, Zhengrong Xu, Chenjie Yu, Xiaoyun Qian, Ling Lu, Shasha Zhang, Yuan Zhang, Wei Xiong, Xia Gao, Zhigang Xu, Renjie Chai

**Affiliations:** ^1^Department of Otolaryngology Head and Neck Surgery, Nanjing Drum Tower Hospital, Nanjing University Medical School, Nanjing, China; ^2^Key Laboratory for Developmental Genes and Human Disease, Ministry of Education, Institute of Life Sciences, Southeast University, Nanjing, China; ^3^Research Institute of Otolaryngology, Nanjing, China; ^4^Co-Innovation Center of Neuroregeneration, Nantong University, Nantong, China; ^5^Shandong Provincial Key Laboratory of Animal Cells and Developmental Biology, School of Life Sciences, Shandong University, Jinan, China; ^6^Shandong Provincial Collaborative Innovation Center of Cell Biology, Shandong Normal University, Jinan, China; ^7^Department of Biotechnology, Federal Urdu University of Arts, Science and Technology, Karachi, Pakistan; ^8^School of Life Sciences, IDG/McGovern Institute for Brain Research, Tsinghua University, Beijing, China; ^9^Jiangsu Province High-Tech Key Laboratory for Bio-Medical Research, Southeast University, Nanjing, China; ^10^Institute for Stem Cell and Regeneration, Chinese Academy of Sciences, Beijing, China

**Keywords:** *Arhgef6*, hair cells, stereocilia, sensorineural hearing loss, guanine nucleotide exchange factors

## Abstract

ARHGEF6 belongs to the family of guanine nucleotide exchange factors (GEFs) for Rho GTPases, and it specifically activates Rho GTPases CDC42 and RAC1. *Arhgef6* is the X-linked intellectual disability gene also known as XLID46, and clinical features of patients carrying *Arhgef6* mutations include intellectual disability and, in some cases, sensorineural hearing loss. Rho GTPases act as molecular switches in many cellular processes. Their activities are regulated by binding or hydrolysis of GTP, which is facilitated by GEFs and GTPase-activating proteins, respectively. RAC1 and CDC42 have been shown to play important roles in hair cell (HC) stereocilia development. However, the role of ARHGEF6 in inner ear development and hearing function has not yet been investigated. Here, we found that ARHGEF6 is expressed in mouse cochlear HCs, including the HC stereocilia. We established *Arhgef6* knockdown mice using the clustered regularly interspaced short palindromic repeat-associated Cas9 nuclease (CRISPR-Cas9) genome editing technique. We showed that ARHGEF6 was indispensable for the maintenance of outer hair cell (OHC) stereocilia, and loss of ARHGEF6 in mice caused HC stereocilia deficits that eventually led to progressive HC loss and hearing loss. However, the loss of ARHGEF6 did not affect the synapse density and did not affect the mechanoelectrical transduction currents in OHCs at postnatal day 3. At the molecular level, the levels of active CDC42 and RAC1 were dramatically decreased in the *Arhgef6* knockdown mice, suggesting that ARHGEF6 regulates stereocilia maintenance through RAC1/CDC42.

## Introduction

Sensorineural hearing loss (SNHL) is the most prevalent sensory defect and affects millions of individuals all over the world (Smith et al., 2005). Various external and internal factors have been shown to contribute to SNHL, including gene mutations, aging, noise exposure, ototoxic medications, and brain tumors, among which genetic factors is responsible of a large number of all cases of deafness (Idan et al., 2013). Most of the hereditary hearing loss belongs to monogenetic disorders. According to the presence of symptoms other than auditory deficits, SNHL is classified as syndromic hearing loss and nonsyndromic hearing loss. More than 400 cases of syndromic hearing loss have been described so far (Toriello et al., 2004).

X-linked intellectual disability (XLID) is a common cause of inherited intellectual disability, affecting around one out of 1000 males (Chiurazzi et al., 2008). XLID is clinically categorized into two subtypes, syndromic X-linked intellectual disability (IDXS) and non-specific X-linked intellectual disability (IDX) (Mulley et al., 1992). IDXS is characterized by multiple defects in organs/tissues in addition to the brain, whereas in IDX patients, intellectual disability is the only clinical symptom. Several IDXS cases have been shown to suffer from hearing deficits, and some of the causative genes have been identified (Cowchock et al., 1985; Gustavson et al., 1993; de Kok et al., 1995; Abidi et al., 1999). *Arhgef6* (also known as *α-PIX* or *Cool-2*) is one of the few genes whose mutations are known to cause XLID (Kutsche et al., 2000). A reciprocal X/21 translocation that breaks the *Arhgef6* transcription at exon 10–11 was shown to be responsible for the severe intellectual disability, mild dysmorphic features, and SNHL in a male patient (Kutsche et al., 2000). Additionally, the IVS1-11T→C mutation in the first intron of *Arhgef6* was found to cause nonspecific mental retardation IDX46 in a large Dutch family (Yntema et al., 1998). This mutation causes skipping of exon 2 and produces a protein presumably lacking 28 amino acids (Kutsche et al., 2000).

GTPases act as molecular switches whose activities are regulated by binding and hydrolysis of GTP (Bourne et al., 1990, 1991). GTPases alternate between the GTP-bound (active) state and GDP-bound (inactive) state, which are facilitated by guanine nucleotide exchange factors (GEFs) and GTPase-activating proteins (GAPs), respectively. The Rho GTPase family consists of around 20 members, including CDC42, RAC1, RhoA, etc., which play important roles in cytoskeletal rearrangements, cell motility, cell polarity, axon guidance, vesicle trafficking, and cell cycle progression (Heasman and Ridley, 2008). *Arhgef6* encodes a protein that belongs to the Rho GEF protein family, and as a Rho GEF, ARHGEF6 activates RAC1 and CDC42, but not RhoA (Manser et al., 1998). In the mouse, two *Arhgef6* transcript variants have been identified, which utilizes different transcription start sites and produces ARHGEF6 protein with slightly different N-termini. Compared with variant 1, variant 2 lacks the amino-terminal calponin homology (CH) domain, but contains the SH3 domain, the Dbl homology (DH) domain, and the pleckstrin homology (PH) domain. The CH domain can bind to Parvins, the SH3 domain can bind to PAKs (p21-activated protein kinases), and the DH and PH domains function as a Rho GEF. Interestingly, the PAK family of kinases are also important downstream effectors of RAC1 and CDC42 (Zhou et al., 2016).

The role of ARHGEF6 in the brain has been investigated using *Arhgef6* knockout mice (Ramakers et al., 2012). In the mouse brain, ARHGEF6 is primarily expressed in the hippocampal neuropil, and loss of ARHGEF6 causes increased dendritic length and spine density but reduced spine synapse numbers in the hippocampus (Ramakers et al., 2012). Furthermore, loss of ARHGEF6 results in reduced long-term potentiation and increased long-term depression in the CA1 hippocampal area, and *Arhgef6* knockout mice show learning and behavioral deficits, suggesting that this mouse model mimics the human XLID phenotypes (Ramakers et al., 2012). At the molecular level, a significant decrease in active RAC1 and CDC42, but not RhoA, was observed in the brain of *Arhgef6* knockout mice (Ramakers et al., 2012). Besides the brain, loss of ARHGEF6 was also shown to affect the immune system (Missy et al., 2008; Korthals et al., 2014). However, the role of ARHGEF6 in the auditory system remains unclear. In this work, we report that ARHGEF6 is expressed in auditory hair cells (HCs) in the mouse cochlea and that loss of ARHGEF6 causes HC stereocilia deficits and progressive HC loss, which eventually leads to hearing loss.

## Materials and Methods

### Ethics Statement

The use of animals and the experimental techniques in this study were performed according to the Animal Care Committee of Southeast University and were approved by the National Institute of Health Guide for the Care and Use of Laboratory Animals.

### *Arhgef6* Knockdown Mice

To generate mutant *Arhgef6* mice with clustered regularly interspaced short palindromic repeat-associated Cas9 nuclease (CRISPR-Cas9) technology, the *Arhgef6* sgRNAs were designed using the CRISPR tool^1^ (Zhang Feng Lab) targeting exon 1 of *Arhgef6*. The reagents and the procedure used for the production of Cas9 mRNA and sgRNA were previously described in detail (Yang et al., 2013; Liu et al., 2017). The T7 promoter was added into the Cas9 coding sequence by PCR amplification using the PX330 vector and the forward and reverse T7-Cas9 primers. The T7-Cas9 PCR products were separated by agarose gel electrophoresis and purified with the QIAquick Gel Extraction Kit (Qiagen, United States). The acquired post-PCR end products were used as the template (500 ng) for *in vitro* transcription using the mMESSAGE mMACHINE T7 Ultra Transcription Kit (Ambion, Thermo Fisher Scientific, United States). Both T7 promoter and targeting sgRNA sequences were added into the sgRNA backbone template by PCR amplification using the forward and reverse T7-sgRNA primers. The PCR product was purified by agarose gel electrophoresis and the QIAquick Gel Extraction Kit (Qiagen) and used as the template (250 ng) for *in vitro* transcription using the MEGA shortscript T7 kit (Ambion, Thermo Fisher Scientific, United States). Both Cas9 mRNA and specific sgRNAs were purified according to the standard protocol by using the phenol:chloroform extraction and alcohol precipitation method and were dissolved in DNase/RNase-free water (Life Technologies). All primer sequences used in this study are listed in **Supplementary Table S1**.

C57BL/6 background mice were chosen as the embryo donors. Cas9 mRNA (50 ng/l), sgRNA-1 (25 ng/μl), and sgRNA-2 (25 ng/μl) targeting the *Arhgef6* gene were mixed and injected into the cytoplasm of the fertilized eggs according to the standard protocols described previously (Yang et al., 2013). The injected embryos were cultured in G1 PLUS medium (10128, Vitrolife) and developed to the two-cell stage *in vitro*. The eggs were then transferred into the oviducts of pseudopregnant female mice.

**FIGURE 1:**
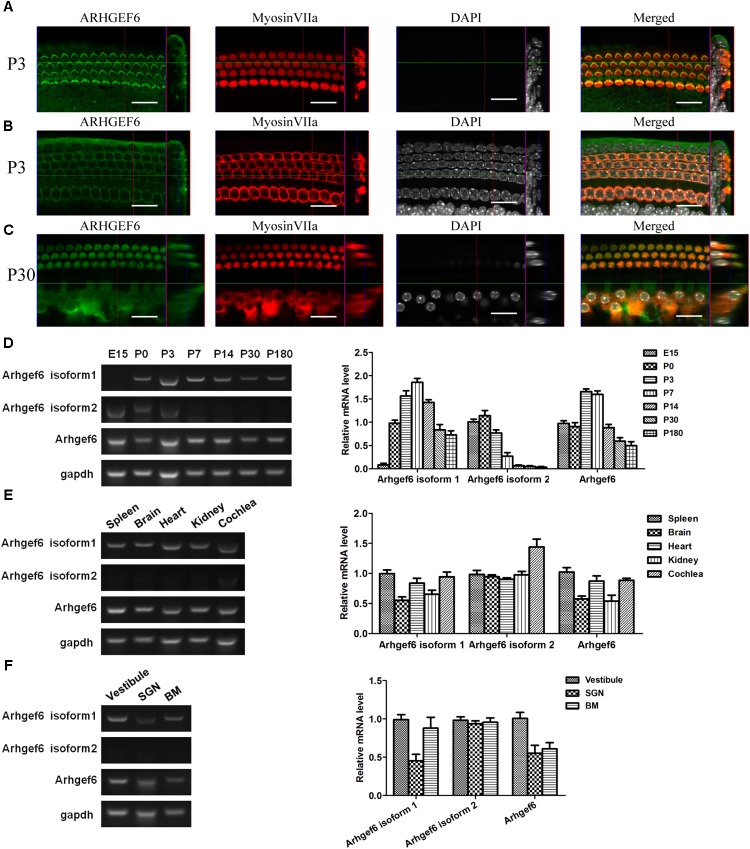
Expression pattern of ARHGEF6 in the cochlea. ARHGEF6 is expressed in the sensory epithelium of the mouse inner ear at P3 **(A,B)** and P30 **(C)**. Scale bar: 20 μm. Expression of *Arhgef6* isoform 1, isoform 2, and *Arhgef6* at different stages including E15, P0, P3, P7, P14, P30, and P180 by RT-PCR and RT-qPCR **(D)**. Expression of *Arhgef6* isoform 1, isoform 2, and *Arhgef6* in different tissues including spleen, brain, heart, kidney, and cochlea by RT-PCR and RT-qPCR **(E)**. Expression of *Arhgef6* isoform 1, isoform 2, and *Arhgef6* in different parts of the cochleae, including the vestibule, basilar membrane, and spiral ganglion by RT-PCR and qRT-PCR **(F)**.

Genomic DNA was extracted from the tails of the newborn pups. To determine the nucleotide sequence of the mutated alleles, the genomic DNA fragment around the sgRNA target site was amplified by PCR using the following two primers: *Arhgef6* forward 5′-CCC ACG TTC CTC TGT TGT CA-3′ and *Arhgef6* reverse 5′-GCA TTT CCA CAA CCA CAG CA-3′. To identify the mutations, DNA sequencing of PCR products was performed after TA cloning into the pMD19T plasmid. After determining the sequences of these pups, one pup was chosen as the F0 mouse because it carried a frameshift mutation due to a 7 bp nucleotide deletion in the *Arhgef6* gene and encoded a truncated version of the protein (**Figures 1A,B**). Offspring of the F0 mouse were genotyped by PCR using the following primers: wildtype *Arhgef6* forward: 5′-TAA ACA GAC TTC TGC CTG GCT CGG T-3′, wildtype *Arhgef6* reverse: 5′-CAA TAA GGT TGT CCT CCT ATC C-3′, mutant *Arhgef6* forward: 5′-CAT AAA CAG ACT TCT TCT TCG G-3′, and mutant *Arhgef6* reverse: 5′-GCA TTT CCA CAA CCA CAG CA-3′.

### Auditory Brainstem Responses (ABR) and Distortion Product Otoacoustic Emissions (DPOAE)

A TDT System III workstation running SigGen32 software (Tucker-Davis Technologies, United States) was used to test mice for close field auditory brainstem response (ABR) and distortion product otoacoustic emission (DPOAE) thresholds as previously described (Chen et al., 2014; Zhu et al., 2015). Mice were injected intraperitoneally with 0.01 g/ml pentobarbital sodium (100 mg/kg body weight) to achieve deep anesthesia. The test was performed in a soundproof room. For the ABR test, three fine-needle electrodes were inserted in the mice at the cranial vertex, underneath the tested ear, and at the back near the tail. ABR tone pips of clicks or 4, 8, 12, 16, 24, 32 kHz stimuli were generated. Auditory thresholds were determined by decreasing sound intensities from 90 to 10 dB until the reaching lowest sound intensity at which the first wave could be recognized. For the DPOAE test, two primary tone frequencies (f1 and f2, with f2/f1 = 1.2 and the f2 level 10 dB greater than the f1level) were given through the earphones, and the sound emission stimulated by the outer hair cells (OHCs) of the tested ear was recorded at the frequency 2f1 – f2 as the f0 wave. Hearing threshold was defined at the lowest sound intensity at which the f0 wave was recognizable.

### RNA Extraction, RT-PCR, and qRT-PCR

Total RNA from different tissues and organs at postnatal day P14, different parts of cochleae at P7, cochleae of different ages, cochlea at P3 and P14 in wildtype and *Arhgef6* knockdown mice were extracted using RNeasy Micro Kits (Qiagen, Valencia, CA, United States) according to the manufacturer’s instructions. The utricle, the basilar membrane (BM), and the modiolus of the cochleae were carefully dissected to determine the mRNA levels in the vestibular sensory cells, the auditory sensory cells, and the spiral ganglion neurons (SGNs), respectively. Then the DNase I (Thermo Fisher Scientific, EN0521) was used for the removal of genomic DNA. The reverse transcription was carried out using the RevertAid First Strand cDNA Synthesis Kit (Thermo Fisher Scientific, K1621) according to the manufacturer’s protocol. PCR using the cDNA template and agarose gel electrophoresis were performed to semi-quantify the mRNA level of *Arhgef6*. The following RT-PCR primers were used: transcript variant 1 of *Arhgef6* forward: 5′- AGAGCGCTCATAAAAGGAAGACAG-3′, transcript variant 1 of *Arhgef6* reverse: 5′-TTGGCCCCCGAATAGAGGT-3′, transcript variant 2 of *Arhgef6* forward: 5′-TGGGGAGCTTGGAGGCACAGAAA-3′, transcript variant 2 of*Arhgef6* reverse: 5′-TTGGCCCCCGAATAGAGGTCATCA-3′, *Arhgef6* forward: 5′-AATCCAGAAGAACGCCTTGTG-3′, *Arhgef6* reverse: 5′-CACTACCCCATTTTTCAGCGA-3′, β*-actin* forward: 5′-ATGGATGATGATATCGCCGCGCTCGTCGTCGACA-3′, and β*-actin* reverse: 5′-CGTAGATGGGCACAGTGTGGGTGACCCCGTCACC-3′. The SYBR Green PCR Master Mix (Roche) was used and the qPCR process were carried out on a BIO-RAD C1000 Touch thermal cycler. The following primers were used for qPCR amplification: CDC42 forward: 5′-CCCATC GGAATATGTACCAACTG-3′, CDC42 reverse: 5′-CCAAGAGTGTATGGCTCTCCAC-3′, RAC1 forward: 5′-ACCGTCTTTGACAACTATTCTGC-3′, RAC1 reverse: 5′-GTCTGTCTGCGGGTAGGAGA-3′, PAK1 forward: 5′-GAAACACCAGCACTATGATTGGA-3′, PAK1 reverse: 5′-GAAACACCAGCACTATGATTGGA-3′, PAK2 forward: 5′-CTGGGGCAAGAGGTTGCTATC-3′, PAK2 reverse: 5′-CAGCGAGGTACTCCATTACCA-3′, PAK3 forward: 5′-CTGAGGATGAACAGTAACAACCG-3′, PAK3 reverse: 5′-CTGGGAAGA TAGAGCGAAGCC-3′, LIMK1 forward: 5′-ATGAGGTTGACGCTACTTTGTTG-3′, LIMK1 reverse: 5′-CTACACTCGCAGCACCTGAA-3′, LIMK2 forward: 5′-GGGCTGTGG CACCTATGTTC-3′, LIMK2 reverse: 5′-CCAGTTGGTGAGGGATTCCTG-3′,COFILIN1 forward: 5′-ATGACATGAAGGTTCGCAAGT-3′, COFILIN1 reverse: 5′-GACAAAAGT GGTGTAGGGGTC-3′, COFILIN2 forward: 5′-GCATCTGGAGTTACAGTGAATGA-3′, COFILIN2 reverse: 5′-CACCAATGTCACCCACCAAGA-3′, ARHGEF6 forward: 5′-AAT CCAGAAGAACGCCTTGTG-3′, ARHGEF6 reverse: 5′-CACTACCCCATTTTTCAGCGA-3′, GAPDH forward: 5′-AGGTCGGTGTGAACGGATTTG-3′, and GAPDH reverse: 5′-TGTAGACCATGTAGTTGAGGTCA-3′.

### Whole Mount Immunostaining

Cochleae from *Arhgef6* knockdown and wildtype mice were dissected and fixed in 4% paraformaldehyde for 1 h at room temperature (RT). Cochleae of adult mice were decalcified in 10% EDTA overnight at 4°C. All of the cochlear samples were carefully dissected. Whole mounts of the BM were washed with 10 mM PBS and blocked with 10% heat-inactivated donkey serum, 1% bovine serum albumin (BSA), 1% Triton X-100, and 0.02% sodium azide in PBS (pH 7.2) for 1 h at RT. Primary antibodies against ARHGEF6 (goat, 1:400 dilution, Santa Cruz Biotechnology) and myosin7a (rabbit, 1:1000 dilution, Proteus Bioscience, 25-6790) were diluted in 5% heat-inactivated donkey serum, 1% BSA, 10% Triton X-100, and 0.02% sodium azide. The samples were incubated with primary antibodies overnight at 4°C. After three washes with 10 mM PBS, the samples were further incubated at RT for 1 h in secondary antibodies (Alexa Fluor 647 or 555 or 488, Invitrogen) diluted in 0.1% BSA and 0.1% Triton X-100. Lastly, samples were again washed with 10 mM PBS five times. Cochleae were imaged with a LSM 700 confocal microscope.

### Scanning Electron Microscopy

Cochleae from *Arhgef6* knockdown and wildtype mice were dissected and then immersed in 2.5% glutaraldehyde in 0.1 M phosphate buffer overnight at 4°C. For mice older than 1 week, the cochleae were decalcified in EDTA for 6 h at RT and then carefully dissected into three turns. The tectorial membrane was removed to expose the hair bundle. The dissected tissues were post-fixed in 1% OsO_4_, dehydrated in a series of ethanol, and dried in a CO_2_ critical-point dryer. Samples were mounted and sputter coated with gold, and stereociliary bundles were examined in all three turns of the cochlea using a Hitachi S-4800 field-emission scanning electron microscope.

### Protein Purification and Western Blot

Cochleae from 10 newborn mice or two adult mice were dissected in cold PBS and lyzed in a homogenizer with 100 μl RIPA lysis buffer (Medium, Hangzhou Fu De Biological Technology) and 1 μl 100× protease inhibitor cocktail (Hangzhou Fu De Biological Technology). The homogenates were centrifuged at 13,000 × *g* at 4°C for 15 min, mixed with 5× SDS-PAGE sample loading buffer (Beyotime Biotechnology), boiled for 15 min, and subjected to SDS-polyacrylamide gel electrophoresis and blotted onto a PVDF membrane. The bound primary antibodies were detected by HRP-conjugated secondary antibodies using the ECL detection system. The following primary antibodies were used: *Arhgef6* monoclonal antibody (rabbit, 1:1,000 dilution, CST), PAK1 polyclonal antibody (rabbit, 1:1,000 dilution, Abcam), phospho-PAK1(Thr423)/PAK2(Thr402) polyclonal antibody (rabbit, 1:500 dilution, CST, 2601), and GAPDH monoclonal antibody (mouse, 1:5,000 dilution, Millipore).

### RAC1 and CDC42 Activation Assay

RAC1 and CDC42 activation assays were performed using a Millipore kit (catalog no. 17-441). Six cochleae from three adult mice for each sample were homogenized for 30 s in Mg^2+^ lysis buffer on ice and centrifuged for 5 min at 13,000 × *g* at 4°C. The supernatant was collected and equally divided into two parts. One half was used for total RAC1 and CDC42 detection and the other half was used for detection of the active forms. For the RAC1 and CDC42 activation assay, protein lysates were incubated with PAK1-bound glutathione agarose beads for 30 min at 4°C. After the beads were washed with the Mg^2+^ lysis buffer, the samples were eluted in SDS loading buffer, boiled for 15 min, and detected by western blot.

### Yeast Two-Hybrid Screen

Yeast two-hybrid screen was performed as described previously (Xu et al., 1998). Since the chicken basilar papilla cDNA library was the only available library and sequences we are working on are evolutionarily conserved between mouse and chicken, cDNA encoding the full-length chicken PAK1 was cloned into the vector pBD-GAL4 Cam (Stratagene). The yeast strain AH109 (Clontech) was transformed sequentially with this bait plasmid and a chicken cochlear cDNA library in the HybriZAP two-hybrid vector. *HIS3* was used as the reporter gene for the screen in the presence of 2.5 mM 3-amino-1,2,4-triazole (3-AT). Positive colonies were further tested for activation of two other reporter genes, *ADE2* and *lacZ*. The prey plasmids in triple-positive yeast colonies were recovered, and cDNA inserts were sequenced.

### Electrophysiology

The cochleae were isolated from P3 wildtype and knockdown mice. The dissection solution contained 141.7 mM NaCl, 5.36 mM KCl, 0.1 mM CaCl_2_, 1 mM MgCl_2_, 0.5 mM MgSO_4_, 3.4 mM L-glutamine, 10 mM glucose, and 10 mM H-HEPES(pH 7.4). The cochlea were then transferred to the recording chamber with recording solution containing 144 mM NaCl, 0.7 mM NaH_2_PO4, 5.8 mM KCl, 1.3 mM CaCl_2_, 0.9 mM MgCl_2_, 5.6 mM glucose, and 10 mM H-HEPES (pH 7.4). The cochleae were used for electrophysiological recording within 1 h. The OHCs in the apical-middle part of the cochleae were recorded. We used an electrophysiology amplifier (HEKA, EPC-10 USB) controlled by the PatchMaster software. Borosilicate glass filaments (Sutter, BF150-117-10) were made with a pipette puller (Sutter, P-2000) and polished with a microforge (Narishige, MF-830) to resistances of 3–5 MΩ. The pipette solution contained 140 mM KCl, 1 mM MgCl_2_, 0.1 mM EGTA, 2 mM MgATP, 0.3 mM Na_2_GTP, and 10 mM H-HEPES (pH 7.2). The hair bundle was deflected with a fluid-jet pipette with a tip diameter of 5–10 μm that was positioned ∼5 μm from the hair bundle to evoke maximum MET currents. The stimulation was in 40 Hz sinusoidal waves delivered from a 27 mm diameter piezoelectric disc driven by a homemade piezo amplifier.

### Statistical Analysis

All of the data are presented as means ± standard errors of the means (SEM) and analyzed by GraphPad Prism 6 software. Statistical significance was determined by two-tailed, unpaired Student’s *t*-test.

## Results

### ARHGEF6 Expression in the Mouse Cochlea

Immunofluorescence staining was performed on whole-mount BMs to examine the spatiotemporal expression of ARHGEF6 in the mouse cochlea. At P3 stage, the earliest time point examined, ARHGEF6 was highly expressed in the stereocilia of cochlear HCs (**Figure 1A**), but lower expressed in the cytoplasm of the HCs (**Figure 1B**). In P30 adult mice, ARHGEF6 was strongly expressed in both the stereocilia and cytoplasm of cochlear HCs (**Figure 1C**). The expression pattern of different transcript variant of *Arhgef6* was detected RT-PCR and qRT-PCR. The expression of *Arhgef6* isoform1 in the cochlea initially increased from embryonic day 15 (E15) to P7, and then decreased from P7 to P180. This expression pattern was similar with total *Arhgef6* detected by *Arhgef6* common primer. *Arhgef6* isoform2 was highly expressed from E15 to P3, and then distinctly decreased at later stage (**Figure 1D**). At P14, the expression of *Arhgef6* isoform1 were observed in spleen, heart and cochlea of wildtype mice. However, at the same stage, *Arhgef6* isoform2 was expressed weakly in different tissues except for cochlea (**Figure 1E**). At P7, *Arhgef6* isoform1 and *Arhgef6* were higher expressed in vestibule and BM than in SGNs, but the expression of *Arhgef6* isoform2 was almost undetectable in vestibule, SGN and BM (**Figure 1F**).

### Generation of the *Arhgef6* Knockdown Mice

**FIGURE 2:**
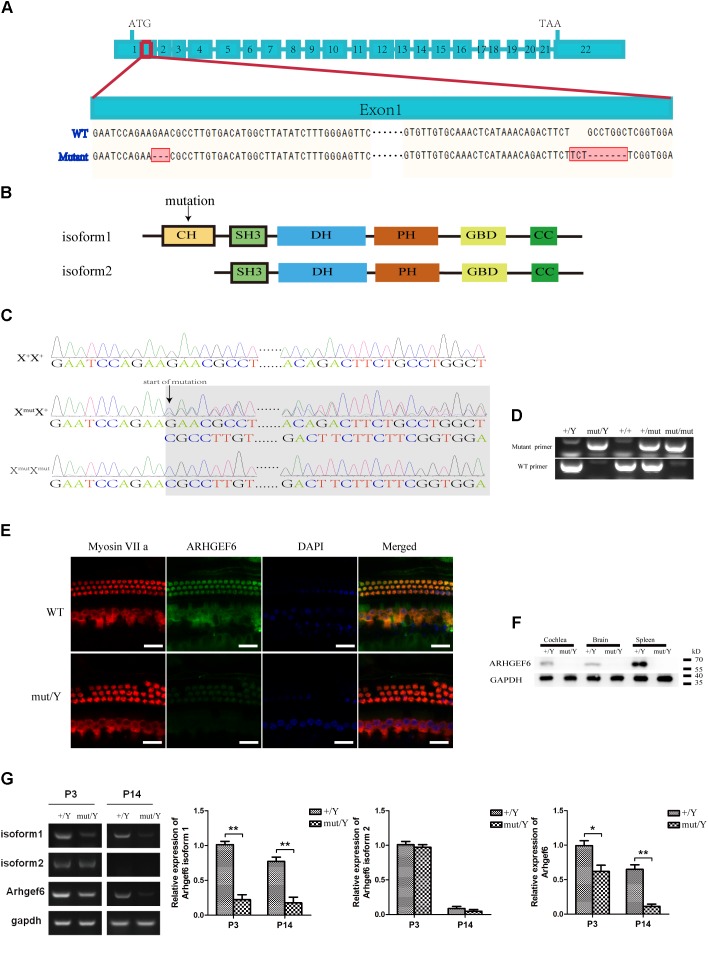
Construction of *Arhgef6* knockdown mice. **(A)** Schematic drawing of the construction method. Targeting sites for *Arhgef6* gene disruption. Exon 1 was disrupted at two sites – the first site has a 3 bp deletion, and the second site has a 3 bp insertion and 7 bp deletion, resulting in a frameshift mutation that codes for a truncated protein. **(B)** Schematic drawing of two isoforms of ARHGEF6 protein. Only isoform 1 was affected by the targeting strategy. **(C)** Sequencing chromatograms of wildtype, heterozygous, and homozygous female mice. **(D)** Genotyping results of wildtype, homozygous, and heterozygous mice by PCR. **(E)** Immunostaining of whole-mount basilar membranes showed strong ARHGEF6 expression in wildtype mice HCs but little expression in knockdown mice HCs at P60. Scale bar: 20 μm. **(F)** Western blot showed that the ARHGEF6 protein can be detected in spleen, brain, and cochlea lysates from wildtype mice but was nearly undetectable in tissue lysates from *Arhgef6* knockdown mice at P30. **(G)** RT-PCR and qRT-PCR showed the expression of *Arhgef6* isoform1, isoform2, and total *Arhgef6* at stages P3 and P14 in wildtype and knockdown mice.

The expression of ARHGEF6 in the HCs suggested that ARHGEF6 might play a role in HC development and/or function. To explore the physiological role of ARHGEF6 in hearing, *Arhgef6* mutant mice were generated using the CRISPR/Cas9 genome-editing technique. The mouse Arhgef6 gene contains 22 exons, with the start and stop codons localizing in the first and last exons, respectively (**Figure 2A**). Two small guide RNAs (sgRNAs) were designed to target sequences after the start codon in exon 1. DNA sequencing showed that a deletion and a replacement were introduced into exon 1, resulting in a total deletion of seven base pairs after the start codon (**Figure 2A**). However, this mutation only occurred in the transcript variant 1 of Arhgef6, but did not occur in transcript variant 2. This change will potentially cause a premature translational stop codon and produce a truncated protein of 95 amino acids as protein isoform 1 but isoform 2 were the same with the wildtype protein (**Figure 2B**). Crossing of F0 female *Arhgef6*^mut/+^ mice with wildtype C57 mice produced four different F1 genotypes, namely *Arhgef6*^+/*Y*^, *Arhgef6*^mut/Y^, *Arhgef6*^+/+^, and *Arhgef6*^mut/+^, which were determined by genotyping PCR and Sanger sequencing (**Figures 2C,D**). Western blot was used to measure the expression of ARHGEF6 in different tissues from control and knockdown mice. ARHGEF6 protein was detected in several tissues from *Arhgef6*^+/*Y*^ mice, but not in *Arhgef6*^mut/Y^ mice (**Figure 2F**). Consistent with this, immunostaining also revealed that ARHGEF6 immunoreactivity was decreased in *Arhgef6*^mut/Y^ mice both at P60 and P3 (**Figure 2E** and **Supplementary Figure S1**).In order to measure the expression of two transcript variants of *Arhgef6* in the mRNA level, the primers for *Arhgef6* isoform1, *Arhgef6* isoform2, and *Arhgef6* common primers were used to further verify the expression of *Arhgef6* nucleotide in *Arhgef6* mutant mice. Both RT-PCR and qRT-PCR results showed that the expression of *Arhgef6* isoform1 and common *Arhgef6* were significantly decreased in mutant mice cochlea at P3 and P14 compared with wildtype mice. However, the difference of *Arhgef6* isoform2 expression between wildtype mice and mutant mice was unobvious at P3 and P14 (**Figure 2G**). These results may suggest a relatively lower percentage of *Arhgef6* isoform2 in wildtype and mutant mice cochlea. Taken together, these results confirmed that we had successfully created *Arhgef6* knockdown mice using the CRISPR/Cas9 genome-editing technique. In the following work, we used *Arhgef6*^mut/Y^ and *Arhgef6*^+/Y^ mice as *Arhgef6* knockdown mice and wildtype controls, respectively.

### *Arhgef6* Knockdown Mice Show Progressive SNHL

**FIGURE 3:**
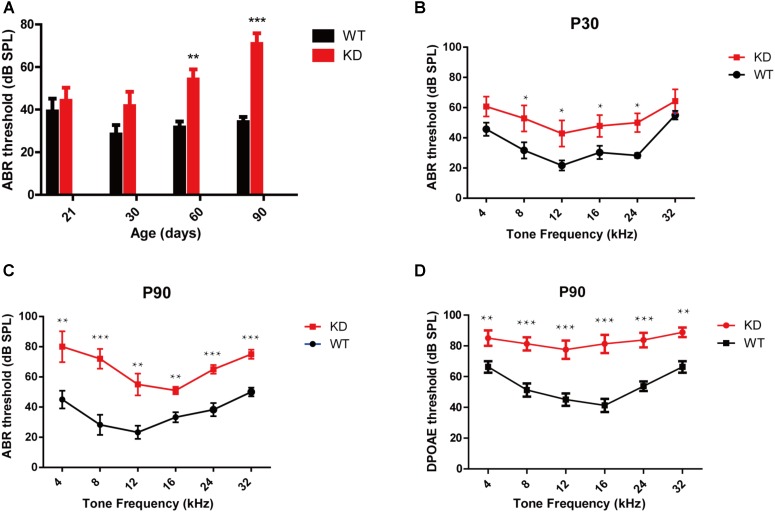
*Arhgef6* knockdown mice show progressive hearing loss. **(A)** The ABR thresholds of wildtype and *Arhgef6* knockdown mice from P21 to P90 in response to click stimuli. **(B)** ABR threshold of wildtype and *Arhgef6* knockdown mice at P30 in response to 4–32 kHz tone pips. **(C)** ABR thresholds of wildtype and *Arhgef6* knockdown mice at P90 in response to 4–32 kHz tone pips. **(D)** The DPOAE thresholds of *Arhgef6* knockdown mice and wildtype mice at P90 in response to 4–32 kHz tone pips. Data are presented as the mean ± SEM, *n* = 10/genotype. ^∗^*p* < 0.05, ^∗∗^*p* < 0.01, and ^∗∗∗^*p* < 0.001.

The auditory function in *Arhgef6* knockdown mice was first evaluated by click stimuli, and progressive SNHL was seen in *Arhgef6* knockdown mice at P21 and P30. The ABR threshold of *Arhgef6* knockdown mice was higher than their wildtype siblings, but the threshold increase was not significant until after P60 (**Figure 3A**). Next we performed pure tone ABR measurements of 4, 8, 12, 16, 24, and 32 kHz stimuli. At P30, the ABR thresholds of *Arhgef6* knockdown mice were generally around 20 dB higher than those of their wildtype siblings, but only at frequencies of 8–24 kHz was there a statistically significant difference (**Figure 3B**). At P90, the ABR threshold difference was significant at all frequencies tested, and the threshold gap increased to 40 dB at frequencies of 4 and 8 kHz (**Figure 3C**). DPOAE was also measured to examine the OHC function of *Arhgef6* knockdown mice at P90. The threshold of *Arhgef6* knockdown mice at P90 was significantly elevated compared to their wildtype siblings, suggesting the dysfunction of OHCs in *Arhgef6* knockdown mice at P90 (**Figure 3D**).

### Stereocilia Morphology Is Affected in *Arhgef6* Knockdown Mice

**FIGURE 4:**
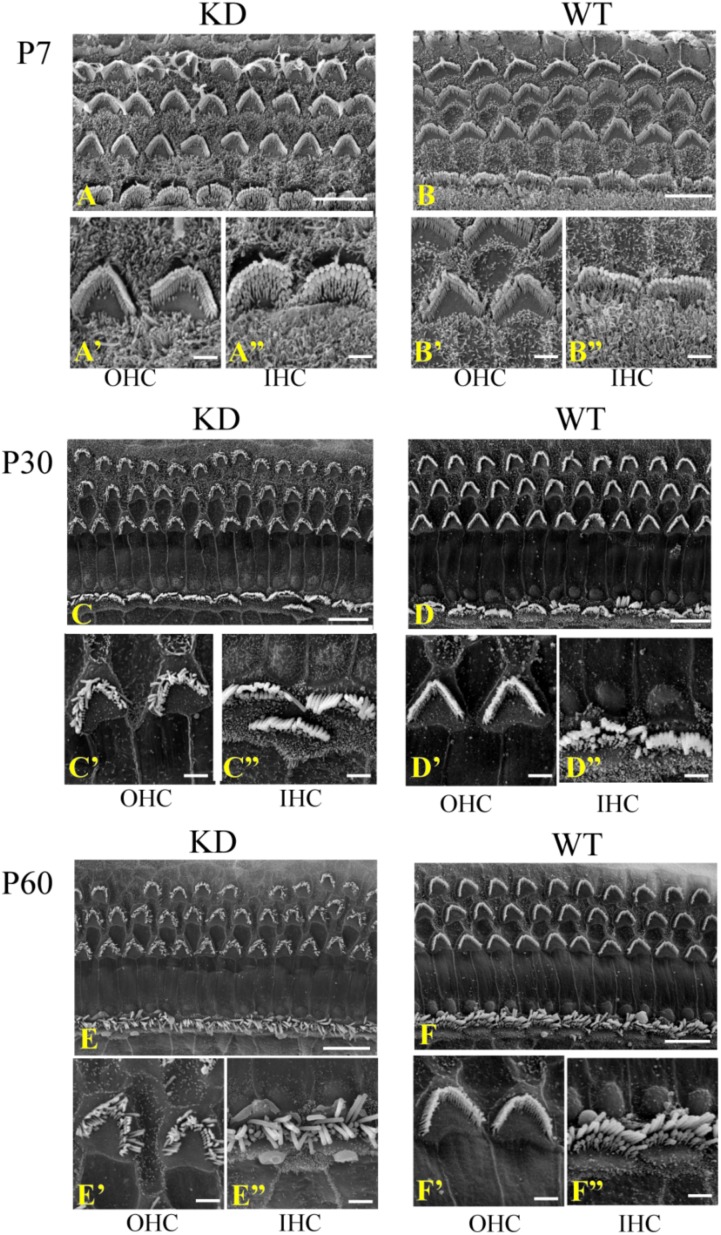
Auditory HC stereocilia morphology is affected in *Arhgef6* knockdown mice at P30 and P60. The SEM image of the middle turn of the cochleae from *Arhgef6* knockdown and wildtype mice at P7 **(A,B)**, P30 **(C,D)**, and P60 **(E,F)**. Scale bars: 10 μm **(A–F)**, 2 μm **(A’–F”)**.

Given the fact that ARHGEF6 is strongly expressed in the stereocilia, we used scanning electron microscopy to examine the ultrastructure of the stereocilia at higher resolution. At P7, the stereocilia morphology of *Arhgef6* knockdown mice was indistinguishable from that of the wildtype control mice (**Figures 4A,B**). At P30, however, the stereocilia of *Arhgef6* knockdown mice were less organized compared to control mice. The OHC stereocilia of *Arhgef6* knockdown mice adopted a splayed morphology, but the inner HC (IHC) stereocilia still had normal morphology (**Figures 4C,D**). By P60, this phenotype was significantly exacerbated and extended to the IHCs, and degeneration of the hair bundles could be observed in most of the OHCs and IHCs (**Figures 4E,F**).

### *Arhgef6* Knockdown Mice Exhibit Distinct OHC Loss

**FIGURE 5:**
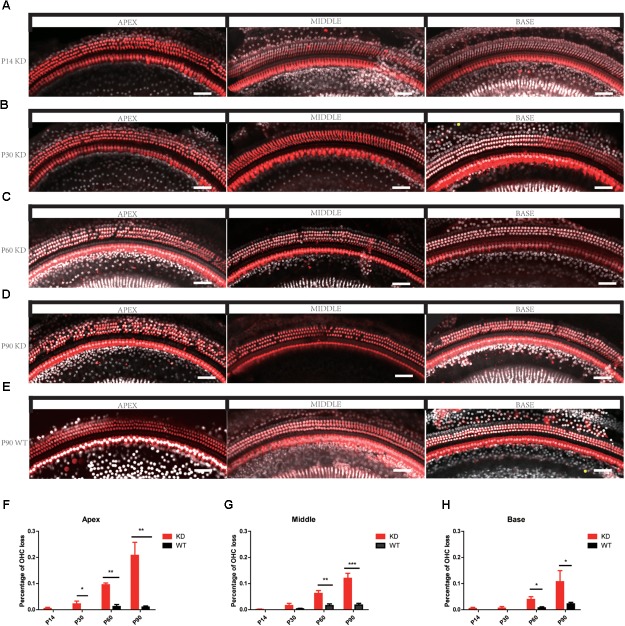
Progressive OHC loss was observed in *Arhgef6* knockdown mice. **(A–E)** Confocal images of whole-mount cochleae from P14/P30/P60/P90 *Arhgef6* knockdown mice and P90 wildtype mice stained with Myosin VIIa (red) and DAPI (white). Scale bar: 20 μm. **(F–H)** The percentage of OHCs lost per 200 μm of the cochlea in *Arhgef6* knockdown mice and wildtype mice at P14/P30/P60/P90 in the apex, middle, and base of the cochlea. Data are presented as the mean ± SEM, *n* = 10/genotype. ^∗^*p* < 0.05, ^∗∗^*p* < 0.01, and ^∗∗∗^*p* < 0.001.

The degeneration of hair bundles indicated that HC loss might occur in *Arhgef6* knockdown mice. We used the HC-specific anti-Myosin VIIa antibody and the nuclear marker DAPI to label HCs in mouse BM whole mounts. No obvious HC loss was seen in P14 *Arhgef6* knockdown cochleae (**Figure 5A**). By P30, a significant amount of scattered OHC loss was seen in the apical turns of *Arhgef6* knockdown cochleae (**Figure 5B**). OHC loss extended from the apical turns to the middle turns of the *Arhgef6* knockdown cochleae by P60, and the OHC loss occurred as a gradient from the apex to the base (**Figure 5C**). The OHC loss was further exacerbated at P90, and also showed a gradient from the apex to the base (**Figures 5D,E**). At all ages examined, HC loss was limited to OHCs, and IHC loss was rarely observed in *Arhgef6* knockdown cochleae. Taken together, these results suggested that *Arhgef6* knockdown mice suffer from progressive OHC loss that started at the apical turn at P30 and extended to all three turns at P60 and P90 (**Figures 5F–H**).To investigate apoptosis after *Arhgef6* knockdown, we used caspase3 staining in *Arhgef6* knockdown and wildtype cochleae and found that *Arhgef6* knockdown cochleae exhibited more caspase3 staining in HCs than wildtype cochleae (**Supplementary Figure S2**).

### Loss of ARHGEF6 Inhibits RAC1/CDC42 Activation

**FIGURE 6:**
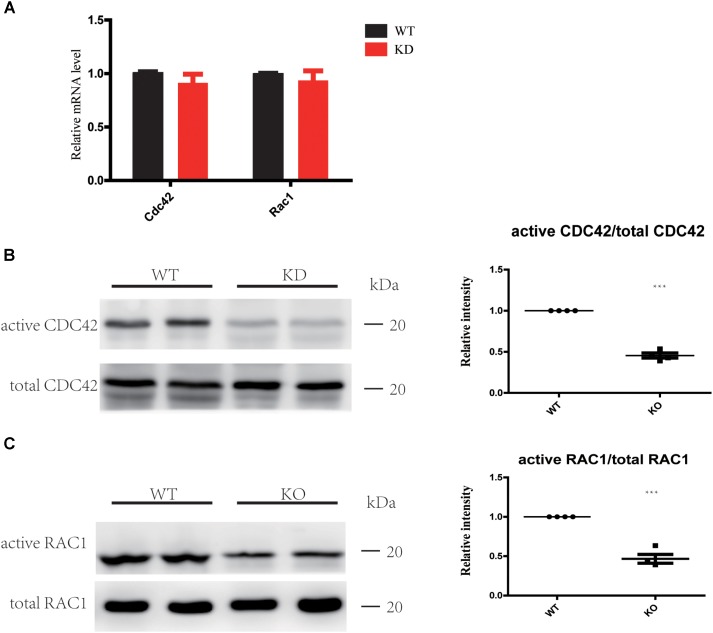
Loss of ARHGEF6 results in decreased levels of active RAC1 and CDC42. **(A)** qPCR measurement of relative RAC1 and CDC42 levels in *Arhgef6* knockdown (KD) mice compared to wildtype mice. **(B,C)** Levels of active and total CDC42 and RAC1 according to the GTPase activity assay and western blot.

The Rho GTPases CDC42 and RAC1 have been shown to play important roles in HC stereocilia development (Grimsley-Myers et al., 2009; Ueyama et al., 2014; Kirjavainen et al., 2015), and the DH domain and PH domain of ARHGEF6 act as a GEF to specially activate CDC42 and RAC1, but not RhoA (Manser et al., 1998). Here we hypothesized that HC degeneration in *Arhgef6* knockdown mice is caused by the decreased levels of activated CDC42 and RAC1. We chose the cochleae of P30 mice as the experimental object. qPCR results showed that the mRNA levels of CDC42 and RAC1 in *Arhgef6* knockdown cochleae were similar to their wildtype littermates, suggesting that the overall expression of CDC42 and RAC1 was not affected in *Arhgef6* knockdown cochleae (**Figure 6A**). We used GTPase activation assays to determine the levels of activated CDC42 and RAC1 in wildtype and *Arhgef6* knockdown cochleae at P30. Western blot revealed significantly reduced active CDC42 and RAC1 protein levels in the *Arhgef6* knockdown cochleae, while the total CDC42 and RAC1 protein levels were not significantly decreased (**Figures 6B,C**). Taken together, these data indicated that the loss of ARHGEF6 results in a significant reduction of active CDC42 and RAC1 in mutant cochleae.

### Loss of ARHGEF6 Results in a Slight Decrease in PAK1 Expression

**FIGURE 7:**
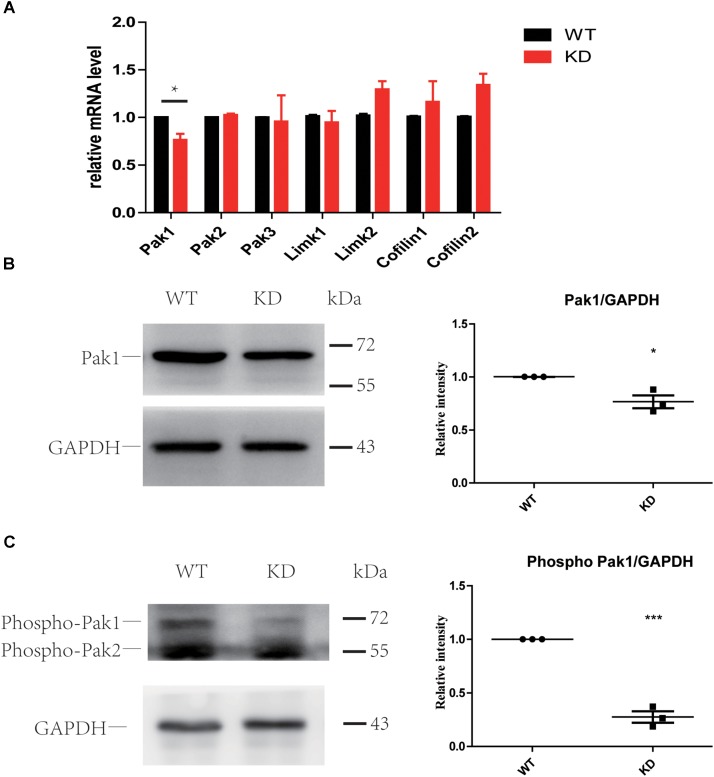
Loss of ARHGEF6 resulted in a significant decrease in PAK1. **(A)** q-PCR results of the relative levels of PAK1, PAK2, PAK3, LIMK1, LIMK2, Cofilin1, and Cofilin2 in the cochleae of *Arhgef6* knockdown mice compared to wildtype mice at P3. Western blot analysis of PAK1 and p-PAK in the cochleae of *Arhgef6* knockdown mice and wildtype mice at P3. PAK1 protein was mildly decreased in *Arhgef6* knockdown cochleae **(B)**, and p-PAK was significantly decreased in the cochleae of *Arhgef6* knockdown mice **(C)**. ^∗^*p* < 0.05, ^∗∗^*p* < 0.01, and ^∗∗∗^*p* < 0.001.

The PAK family of kinases is important downstream effectors of RAC1 and CDC42 that can also bind to ARHGEF6 and ARHGEF7 through the SH3 domain and can modulate their activity independently or can help to coordinate the Rac/Cdc42 signaling (Manser et al., 1998; Bagrodia and Cerione, 1999; Manser and Lim, 1999; Govek et al., 2011). Many studies have reported that PAKs can also act as activators of ARHGEF6 by binding to the SH3 domain of ARHGEF6 and modulating its activity independently or by helping to coordinate RAC/CDC42 signaling (Manser et al., 1998; Chan et al., 2008; Lucanic and Cheng, 2008; Valdes et al., 2011; Santiago-Medina et al., 2013; Radu et al., 2014). Active RAC1 and CDC42 can phosphorylate PAKs and further influence LIMKs and Cofilin through PAKs (Scott and Olson, 2007). Thus, we examined the PAK-LIMK-Cofilin pathway in wildtype and *Arhgef6* knockdown cochleae at P30. qPCR results showed that only PAK1 was significantly decreased in *Arhgef6* knockdown cochleae, while PAK2, PAK3, and LIMK1 were comparable in *Arhgef6* knockdown and wildtype cochleae and LIMK2, Cofilin1, and Cofilin2 were slightly increased in *Arhgef6* knockdown cochleae, but these increases were not statistically significant (**Figure 7A**). Western blot results also showed decreased levels of PAK1 in *Arhgef6* knockdown cochleae (**Figure 7B**), and there were decreased levels of p-PAK1, which is the active form of PAK1, in the *Arhgef6* knockdown cochleae (**Figure 7C**). This result was consistent with decreased levels of active RAC1 and CDC42 in *Arhgef6* knockdown cochleae. In an effort to identify PAK1 binding partners in the inner ear, we carried out yeast two-hybrid screens of a chicken cochlear cDNA library using PAK1 as bait (**Supplementary Table S2**). All of the identified positive clones encoded ARHGEF6 and ARHGEF7, suggesting that these two proteins are strong PAK1 binding partners in the cochlea.

### Loss of ARHGEF6 Did Not Affect the Synapse Density in Cochlear IHCs

**FIGURE 8:**
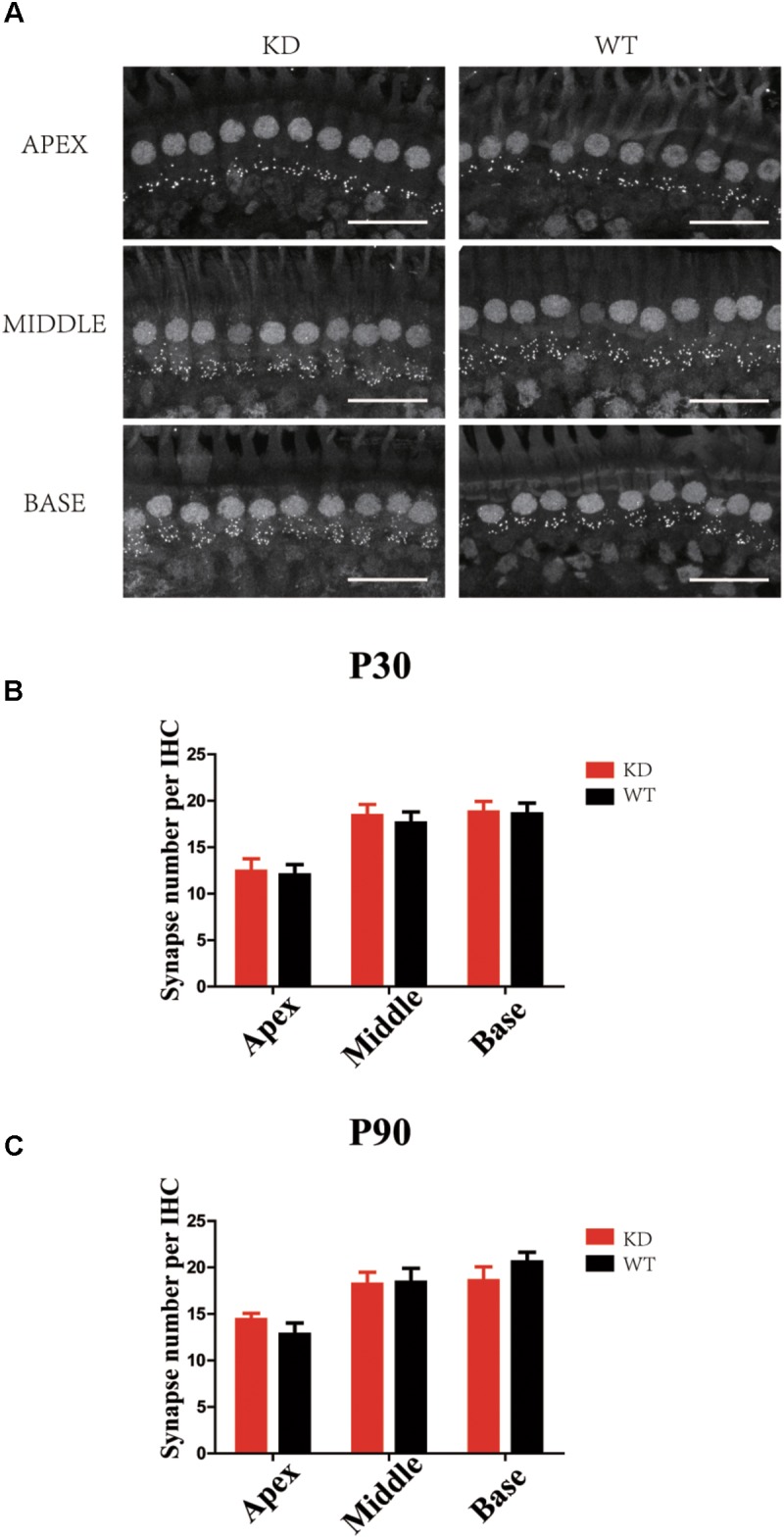
Synapse staining by Ctbp2 in *Arhgef6* knockdown cochleae is similar to wildtype cochleae. **(A)** Confocal images of whole-mount cochleae in P30 *Arhgef6* knockdown mice and wildtype mice stained with the presynaptic marker Ctbp2. Scale bar: 20 μm. The number of synapses stained by Ctbp2 per IHC in the three turns of the cochlea in *Arhgef6* knockdown and wildtype mice at P30 **(B)** and P90 **(C)**. Data are presented as the mean ± SEM, *n* = 10/genotype.

The RAC-PAK-LIMK-Cofilin signaling pathway has been reported to regulate synaptic function and spine morphology mainly through depolymerization of F-actin (Meng et al., 2002, 2003; Zhang et al., 2005). To investigate whether loss of ARHGEF6 affects the synapse density in HCs, we used the anti-Ctbp2 antibody to label the ribbon synapse in the IHCs of the mouse cochlea. The synapse density of IHCs in P30 *Arhgef6* knockdown cochleae was unchanged compared to controls (**Figures 8A,B**), and at P90, there was still no significant difference in the synapse density between *Arhgef6* knockdown cochleae and wildtype controls (**Figure 8C**), suggesting that synapse density in cochlear HCs was not affected by *Arhgef6* disruption.

### Loss of ARHGEF6 Did Not Affect the Excitable Currents in OHCs of Neonatal Mouse Cochleae

**FIGURE 9:**
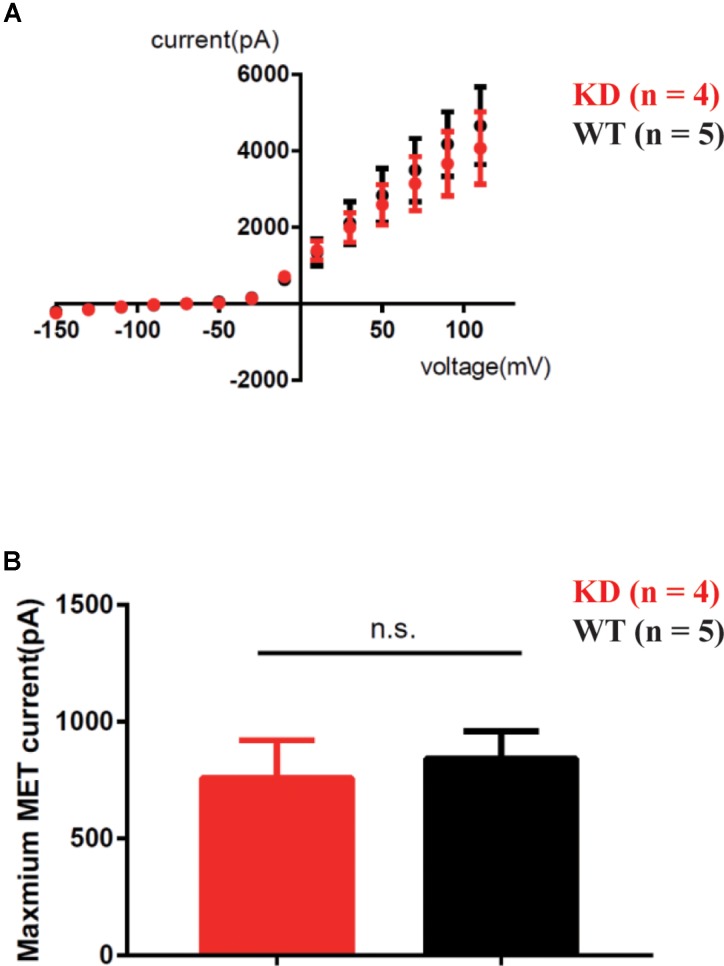
MET was not affected in *Arhgef6* knockdown mice at P3. **(A)** IV curves were recorded from OHCs. The holding potential was altered from -150 to 110 mV in 20 mV steps. **(B)** Statistical analysis showed no obvious difference in maximum MET current between wildtype and *Arhgef6* knockdown OHCs. The number of cells for each experiment is indicated.

To determine whether loss of ARHGEF6 affects the membrane potential currents of OHCs, we used the whole-cell patch-clamp technique to measure the voltage-gated and mechanically gated currents of OHCs in *Arhgef6* knockdown cochleae at P3. The IV curve was not significantly changed in the *Arhgef6* knockdown OHCs compared to wildtype OHCs (**Figure 9A**), and the evoked maximal mechanoelectrical transduction (MET) current was well-preserved in *Arhgef6* knockdown OHCs (**Figure 9B**). Together, these results suggest that the OHCs in *Arhgef6* knockdown cochleae had normal MET currents. Given the fact that bundle degeneration is not apparent until P30, it is not surprising that OHC MET currents are normal at P3 before the cochlea fully matures.

## Discussion

In the present work, we first created the *Arhgef6* knockdown mice in which the mutation only affect *Arhgef6* isoform1 but cannot affect *Arhgef6* isoform2. In *Arhgef6* knockdown mice, expression of *Arhgef6* isoform1 is completely lost due to premature termination in protein translation, and the complete loss of functional *Arhgef6* isoform1 often results in a great loss of total *Arhgef6* especially after P14 considering a relatively low percentage of *Arhgef6* isoform2 in adulthood. We show that disruption of *Arhgef6* decreases the activity of the Rho GTPases RAC1/CDC42 and decreases the activity of PAK1 in the cochlea at P30. Loss of ARHGEF6 causes stereocilia development deficits and eventually leads to progressive HC loss and hearing loss in mice. Rho GTPases are molecular switches whose activity is determined by the association with or hydrolysis of GTP. Because of their wide expression, specific roles in actin polymerization, and versatility as signal transducers, Rho GTPases were proposed to play important roles in HC stereocilia development (Kollmar, 1999). *Rac1* and *Rac3* transcripts are detected in the developing cochlea (Grimsley-Myers et al., 2009), and *RAC1* disruption in mice causes severely shortened cochleae with reduced numbers of auditory HCs. Moreover, *RAC1*-deficient HCs show stereocilia disorganization and planar cell polarity (PCP) defects (Grimsley-Myers et al., 2009). No obvious morphological defects are observed in *Rac3* knockout mice; however, *Rac1*/*Rac3* double knockout mice display enhanced vestibular and cochlear malformations compared to *RAC1* knockout mice (Grimsley-Myers et al., 2012). CDC42 has been shown to localize in the stereocilia, and the stereocilia of *Cdc42* knockout mice develop normally but progressively degenerate after maturation, resulting in progressive hearing loss (Ueyama et al., 2014). Similar to *Rac1*, *Cdc42* disruption in mice also results in PCP deficits (Kirjavainen et al., 2015).

ARHGEF6 is a Rho-GEF that specifically activates RAC1 and CDC42 (Manser et al., 1998). We found that ARHGEF6 is expressed in HC stereocilia in mice and that loss of ARHGEF6 leads to the decreased activity of CDC42 and RAC1, which results in stereocilia disorganization and progressive HC loss and subsequent hearing loss. It is notable that the inner ear phenotypes of *Arhgef6* knockdown mice are less severe than those of *Rac1* or *Cdc42* knockout mice. Neither PCP deficits nor cochlear shortening are observed, and HC loss is less severe and is limited to OHCs in *Arhgef6* knockdown mice. The relatively weak inner ear phenotypes might be explained by the fact that active RAC1 and CDC42 are reduced, but not abolished, in the *Arhgef6* knockdown mice. The level of active RAC1/CDC42 in *Arhgef6* knockdown mice is about the half of that of control mice, suggesting that other GEFs might also activate RAC1/CDC42 in the inner ear.

One possible candidate is DOCK4, which is a Rho-GEF that activates Rap GTPase (Yajnik et al., 2003). Interestingly, a novel isoform of DOCK4 (DOCK4-Ex49) was found to be specifically expressed in the brain, eye, and inner ear and to strongly activate RAC GTPase (Yan et al., 2006). Moreover, DOCK4-Ex49 was shown to bind to the Usher protein harmonin and to localize in the HC stereocilia (Yan et al., 2006). Another candidate is ARHGEF7 (also known as β-PIX or Cool-1), which is a homolog of ARHGEF6 that also activates RAC1 and CDC42 (Bagrodia et al., 1998; Manser et al., 1998). *Arhgef7* mRNA expression has been detected in FACS-sorted HCs by RNA sequencing (Scheffer et al., 2015; Shen et al., 2015), and consistent with this ARHGEF7 was detected in a proteomic screening of mouse vestibular hair bundle proteins (Krey et al., 2015). Further investigation is needed to determine which GEF or GEFs are responsible for the remaining RAC1/CDC42 activity in *Arhgef6* knockdown cochleae.

PAK1 is an important downstream effecter of both RAC1 and CDC42 (Bagrodia and Cerione, 1999; Manser and Lim, 1999). Active PAKs can enhance the phosphorylation of the LIM kinase family (LIMK1 and LIMK2), which in turn decreases the activity of Cofilins, which affect the growth of actin filaments and the formation of pseudopodia. Therefore, the PAK-LIMK-Cofilin signaling pathway has been proposed to be involved in the process of cell adhesion and motility (Zhang et al., 2011; Tong et al., 2018). The RAC-PAK signaling pathway has been reported to affect basal body positioning and stereocilia morphogenesis (Sipe and Lu, 2011; Sipe et al., 2013), and molecular inhibitors of RAC and PAK can cause positional defects in cochlear explants (Sipe and Lu, 2011). In the mammalian central nervous system, LIMK-Cofilin signaling is important in regulating the actin cytoskeleton, spine morphology, and synaptic function (Meng et al., 2003, 2004). ARHGEF6 co-localizes with PSD95 in dendritic spines in hippocampal slice cultures, and knockdown of ARHGEF6 causes abnormalities in spine morphology and synaptic density in the hippocampus both in vivo and in vitro (Node-Langlois et al., 2006; Ramakers et al., 2012). However, the synapse density of IHCs was not significantly affected by loss of ARHGEF6. The levels of Cofilin1 and Cofilin2 were slightly increased, and this might compensate, at least to some degree, for the decreased levels of PAK1 and p-PAKs in the *Arhgef6* knockdown cochlea.

*Arhgef6* is the eighth MRX gene identified to cause XLMR and the third MRX gene associated with Rho GTPases (Kutsche et al., 2000). The proband with a reciprocal X/21 translocation that affects ARHGEF6 expression shows SNHL in addition to intellectual disability, while hearing deficit was not reported in the Dutch family with the *Arhgef6* IVS1-11T→C mutation (Yntema et al., 1998; Kutsche et al., 2000). However, *Arhgef6* knockdown led to hearing loss in this work. The difference in *Arhgef6* gene mutations might account for the discrepancy in the phenotypes. The reciprocal X/21 translocation in the proband occurs in intron 10 of the *Arhgef6* gene, which interrupts the expression of functional ARHGEF6 (Kutsche et al., 2000). Similarly, in *Arhgef6* knockdown mice, expression of *Arhgef6* isoform1 is completely lost and expression of *Arhgef6* isoform2 were nearly negligible after P14. So the *Arhgef6* KD mice exhibit progressive hearing loss. The *Arhgef6* IVS1-11T→C mutation, however, results in preferential skipping of exon 2, which will potentially produce a protein lacking 28 amino acids (Kutsche et al., 2000). This truncated protein might still function in some manner in the inner ear, but not in the brain, and thus cause intellectual disability but not hearing loss in patients or it might cause age-related hearing loss with no symptoms at a young age. Further investigation is needed to fully address this question.

## Conclusion

In summary, we found that ARHGEF6 is expressed in the HCs of the mouse cochlea and that loss of ARHGEF6 inhibits the activation of the Rho GTPases CDC42/RAC1, which causes HC stereocilia disorganization. Thus, ARHGEF6 loss leads to progressive HC loss and subsequent hearing loss. Our findings identify *Arhgef6* as a potential syndromic hearing loss gene that might prove useful for clinical diagnosis.

## Author Contributions

RC and XG conceived the project. ZhiX and RC designed the experiments. ZheX and YW developed the *Arhgef6* knockdown mice and conducted the yeast two-hybrid screen. WX, SZ, and SL carried out the electrophysiological assays. CZ and CC carried out the scanning electron micrographs. CZ, WZ, XQ, CY, BS, YZ, and LL performed the rest of the experiments. CZ, ZZ, XY, ZheX, and QH analyzed the data. CZ, RC, WM, and ZhiX wrote the manuscript.

## Conflict of Interest Statement

The authors declare that the research was conducted in the absence of any commercial or financial relationships that could be construed as a potential conflict of interest.

## Footnotes

1 http://crispr.mit.edu/
