# Supplementary material for: Loss of ARHGEF6 Causes Hair Cell Stereocilia Deficits and Hearing Loss in Mice
Source: Front Mol Neurosci. 2018 Oct 2;11:362. doi: 10.3389/fnmol.2018.00362 (PMC6176010; doi:10.3389/fnmol.2018.00362)

Supplementary Material

**Loss of Arhgef6 Causes Hair Cell Stereocilia Deficits and Hearing Loss in Mice**

**Che****ngwen Zhu^1,2,3#^, Cheng Cheng^1,2,3,4#^, Yanfei Wang^5,6^, Muhammad Waqas^2,7^, Shuang Liu^8^, Weijie Zhu^2^, Buwei Shao^2^, Zhong Zhang^2^, Xiaoqian Yan^2^, Qingqing He^1^, Zhengrong Xu^1^, Chenjie Yu^1^, Xiaoyun Qian^1^, Ling Lu^1^, Shasha Zhang^2,3,4,9^, Yuan Zhang^2^, Wei Xiong^8^, Xia Gao^1,3^*, Zhigang Xu^5,6^*, Renjie Chai^2,3,4,9,10^***

^1^ Department of Otolaryngology Head and Neck Surgery, Affiliated Drum Tower Hospital of Nanjing University Medical School, Nanjing 210008, China

^2^ Key Laboratory for Developmental Genes and Human Disease, Ministry of Education, Institute of Life Sciences, Southeast University, Nanjing 210096, China

^3^ Research Institute of Otolaryngology, Nanjing 210008, China

^4^ Co-Innovation Center of Neuroregeneration, Nantong University, Nantong 226001, China

^5^ Shandong Provincial Key Laboratory of Animal Cells and Developmental Biology, Shandong University School of Life Sciences, Qingdao, Shandong 266237, China

^6^ Shandong Provincial Collaborative Innovation Center of Cell Biology, Shandong Normal University, Jinan, Shandong 250014, China

^7^ Department of Biotechnology, Federal Urdu University of Arts, Science and Technology, Gulshan-e-Iqbal campus, Karachi, Pakistan

^8^ School of Life Sciences, IDG/McGovern Institute for Brain Research, Tsinghua University, Beijing 100084, China

^9^ Jiangsu Province High-Tech Key Laboratory for Bio-Medical Research, Southeast University, Nanjing 211189, China

^10^ Institute for Stem Cell and Regeneration, Chinese Academy of Sciences, Beijing 100101, China

^#^ These authors contributed equally to this work.

**Correspondence:** Renjie Chai: renjiec[@seu.edu.cn](mailto:lihuawei63@gmail.com)

Zhigang Xu: xuzg@sdu.edu.cn

Xia Gao: xiagao@aliyun.com

**Supplementary Table1**

Primer sequences of T7-cas9 and T7-sgRNA

| T7-sgRNA-1 | F | TAATACGACTCACTATAGGCATGTCACAAGGCGTTCTTCGTTTTAGAGCTAGAAATAGC |
| --- | --- | --- |
|  | R | AAAAGCACCGACTCGGTGCC |
| T7-sgRNA-2 | F | TAATACGACTCACTATAGGCTTACcttctccaccgagccGTTTTAGAGCTAGAAATAGC |
|  | R | AAAAGCACCGACTCGGTGCC |
| T7-Cas9 | F | TAATACGACTCACTATAGGGAGAATGGACTATAAG GACCACGAC |
|  | R | GCGAGCTCTAGGAATTCTTAC |

**Supplementary Table 2**

**PAK1-binding partners identified from yeast two-hybrid screening.** A fragment containing the full length of PAK1 was used as bait to screen a chicken cochlear cDNA library.

GenBank accession No. Protein Prey redundancy

NM_001006432 ARHGEF6 19

XM_004938547 ARHGEF7 4

# Supplementary Figure 1

Immunostaining of whole-mount basilar membranes showed strong ARHGEF6 expression in wild-type mouse HCs especially in sterocilia but little expression in knockdown mouse HCs at P3. Scale bar: 20 μm.


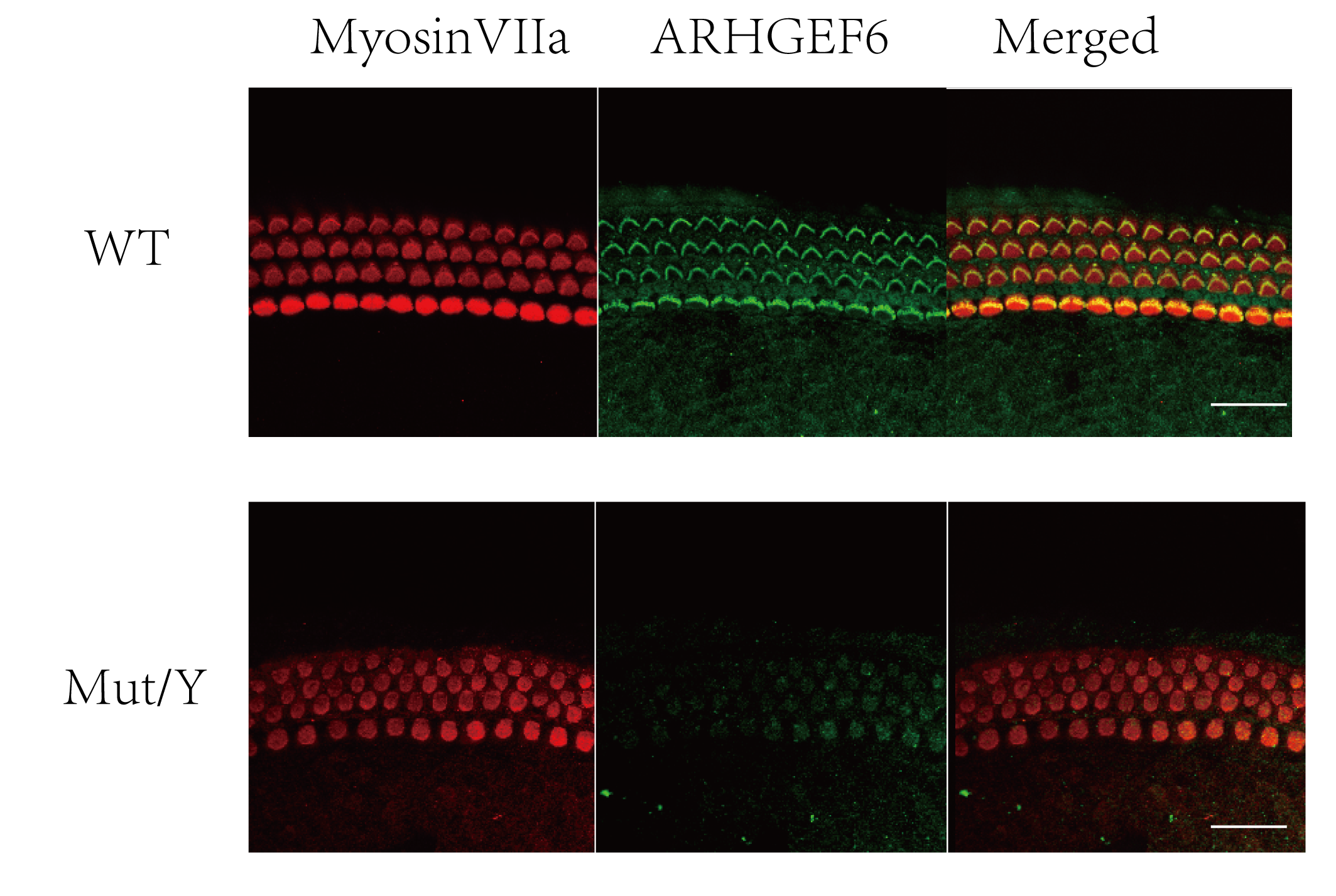


# Supplementary Figure 2

Detection of apoptosis by cleaved caspase3 marker at P30. The expression of caspase3 is stronger in *Arhge6* knockdown mice than their wildtype littermates. Scale bar: 20 μm.


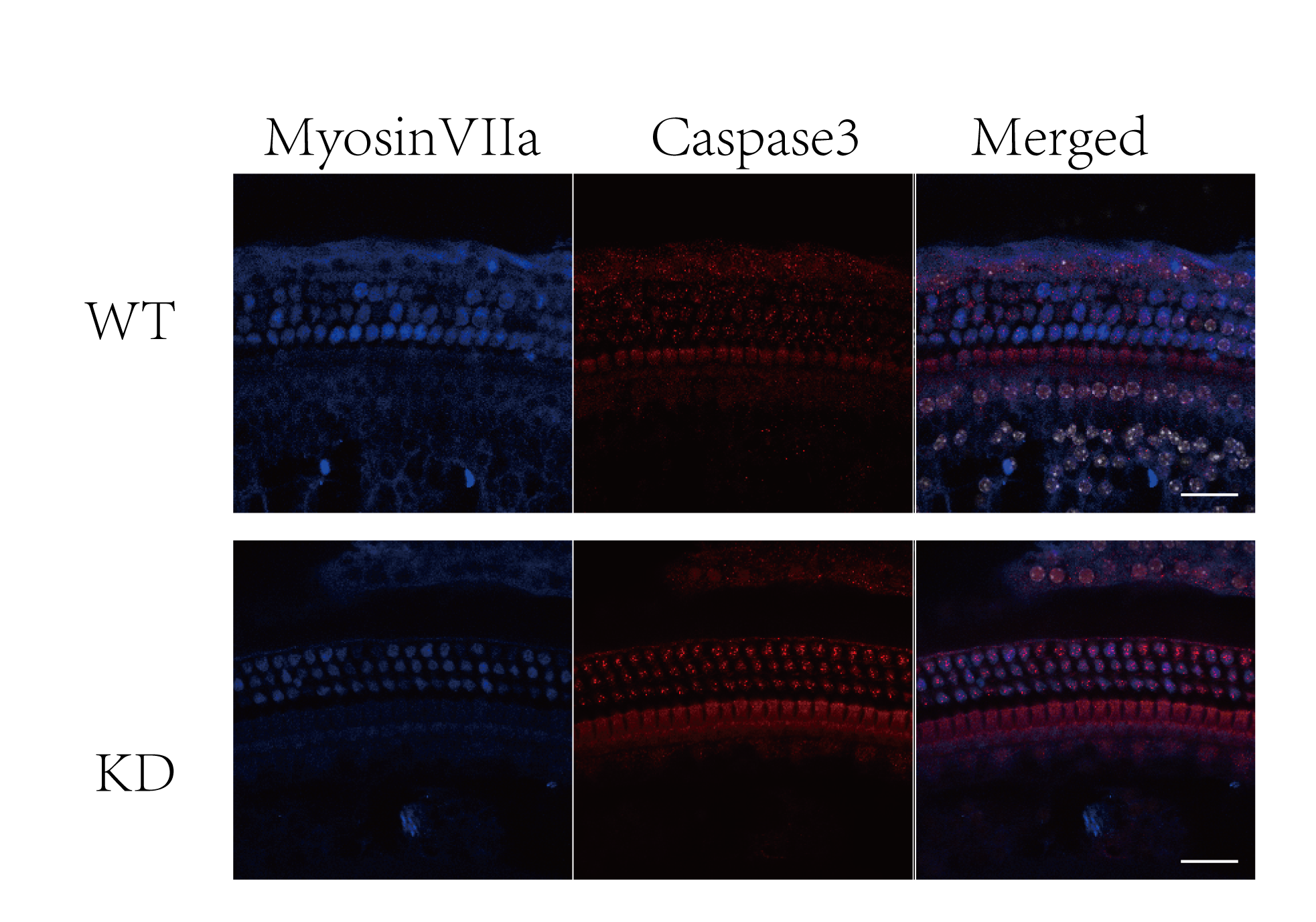

Supplement: Supplementary file 1 [file Data_Sheet_1.docx]
